# Supplementary material for: Genomics and Antimicrobial Susceptibility of Clinical Pseudomonas aeruginosa Isolates from Hospitals in Brazil
Source: Pathogens. 2023 Jul 8;12(7):918. doi: 10.3390/pathogens12070918 (PMC10384983; doi:10.3390/pathogens12070918)
Supplement: Supplementary file 1 [file pathogens-12-00918-s001.zip › Supplementary Table S1. Serotypes and ST.pdf]

Supplementary Table S1. Heatmap showing the distribution of predicted serotypes according to ST.

ST	Predicted serotype												Total
	O1	O2	O3	O3 O6	O4	O5	O6	O7	O9	O10	O11	O12	
10	1												1
111												1	1
179							3						3
183						1							1
207	1												1
234					1								1
235											16		16
238	1												1
244						1							1
252	3												3
253										1			1
261							1						1
274			7										7
277		3				1							4
282							1						1
309											3		3
313	3												3
316											1		1
381		3				1							4
385							1						1
389					2								2
446											5		5
498	1												1
554						1							1
557								3					3
591						1							1
598									2				2
633							1						1
640											1		1
644											1		1
697											1		1
709						1							1
803			1										1
875							2						2
973											1		1
1086								1					1
1122											1		1
1129							1						1
1212											1		1
1284											1		1
1290									1				1
1560					1								1

1600			1										1
1717							1						1
1748							1						1
1966							1						1
2234							1						1
2238							1						1
2317						2							2
2375	1												1
2475		1											1
2524											1		1
2629											1		1
3142					1								1
3214						1							1
3267							1						1
3494										1			1
4022											1		1
4072			1										1
**Δ** mutL				1									1
4083*										1			1
4084*							1						1
4085*											1		1
4086*											1		1
4087*											1		1
4088*									4				4
4089*	1												1
4090*		1											1
4113*						1							1
4114*											1		1
4115*											1		1
Total	12	8	10	1	5	11	17	4	7	3	40	1	119
%	10.1	6.7	8.4	0.8	4.2	9.2	14.3	3.4	5.9	2.5	33.6	0.8	100
Predicted serotype	O1	O2	O3	O3 O6	O4	O5	O6	O7	O9	O10	O11	O12	Total
